# Association between depression and the risk for fracture: a meta-analysis and systematic review

**DOI:** 10.1186/s12888-018-1909-2

**Published:** 2018-10-17

**Authors:** Lei Qiu, Qin Yang, Na Sun, Dandan Li, Yuxin Zhao, Xiaotong Li, Yanhong Gong, Chuanzhu Lv, Xiaoxv Yin

**Affiliations:** 1 School of Public Health, Tongji Medical College, Huazhong University of Science and Technology, 430030 Wuhan, People’s Republic of China; 2 School of Management, Hainan Medical University, Haikou, People’s Republic of China; 3 Department of Emergency, The Second Affiliated Hospital of Hainan Medical University, Haikou, 571199 People’s Republic of China; 4 Emergency and Trauma College, Hainan Medical University, Haikou, 571199 People’s Republic of China

**Keywords:** Depression, Fracture, Meta-analysis

## Abstract

**Background:**

Several studies have suggested that depression is associated with an increased risk for fracture; however, the results are conflicting. This study aimed to conduct a meta-analysis of cohort studies assessing the association between depression and the risk for fracture.

**Methods:**

Relevant studies were identified by a search of Web of Science, PubMed, Embase, China National Knowledge Infrastructure and WanFang database to Feb 2018. Cohort studies on the relationship between depression and the risk for fracture in the general population were included in the meta-analysis. Data collection was in accordance with the Meta-analysis of Observational Studies in Epidemiology (MOOSE) guidelines, and the quality of the included studies was assessed using the Newcastle–Ottawa scale. Two independent investigators screened the abstracts and full texts of the studies, extracted data, and assessed the quality of the study. Either a fixed-effect or random-effects model was used to compute the pooled risk estimates when appropriate.

**Results:**

In total, 16 cohort studies with 25 independent reports that included 414,686 participants during a follow-up duration of 3–14 years were included in the analysis. The pooled hazard ratio (HR) for total fracture was 1.24 (95% confidence interval [CI]: 1.14–1.35; *P* < 0.001 for heterogeneity; random-effects model). In the subgroup analyses conducted in terms of study region, the pooled HR for the studies conducted in Europe was higher (HR: 1.76; 95% CI: 1.44–2.17; *P* = 0.792 for heterogeneity) than that in America and Asia, with a significant difference between the groups (*P* = 0.036).

**Conclusion:**

The results of our meta-analysis suggest that depression is prospectively associated with a significantly increased risk for fracture, which may have substantial implications, both clinical and preventive.

**Electronic supplementary material:**

The online version of this article (10.1186/s12888-018-1909-2) contains supplementary material, which is available to authorized users.

## Background

Osteoporotic fracture is a critical health problem worldwide because it causes severe pain, disability, decreased quality of life, and increased mortality and health costs [1, 2]. More than one-third of women and approximately one-tenth of men aged 50 years will sustain a major osteoporotic fracture in their remaining lifetime [3]. Similar to fracture, depression was a common disorder in modern society, with a high prevalence among the general population [4]. The lifetime incidence of depression is approximately 16% among adults in the United States [5]. Globally, the total number of individuals with depression was around 300 million in 2015, which is equivalent to 4.4% of the world’s population [6].

During the past decades, several studies have assessed the association between depression and the risk for fracture. However, the results were conflicting [7–9]. Some studies have reported that depression, was often complicated with decreased bone mineral density (BMD) and bone loss and is associated with a significant increased risk in fracture, but other studies have not found such risk. A previous meta-analysis pooled results from 10 studies published before 2009 as a secondary analysis and showed the association between depression and an increased risk for fracture [10]. However, this previous study had several limitations. First, the potential sources of heterogeneity were not explored, with limited description on heterogeneity across studies. Second, its subgroup analysis was limited to sex and whether the study controlled for the use of antidepressants and lack of subgroup analyses stratified by other important study and participant characteristics. For example, because the prevalence of depression varied in different regions, investigating the geographical region differences in the depression–fracture association is of interest. In addition, more studies conducted in various countries have been published in recent years, which allowed for a more detailed analysis of the relationship between depression and the risk for fracture. Given the limitations of previous review and the recent publication of numerous large cohort studies, it is necessary for us to assess the effect of depression on the risk for fractures via an updated meta-analysis based on cohort studies.

## Methods

### Search strategy

This review was conducted in accordance with the Meta-analysis of Observational Studies in Epidemiology (MOOSE) guidelines [11], with reference to the Preferred Reporting Items for Systematic Reviews and Meta-analyses (PRISMA) [12]. A literature search on prospective or retrospective cohort studies showing the association between depression and fracture in Web of Science, PubMed, Embase, China National Knowledge Infrastructure (CNKI) and WanFang database was conducted from inception to February 2018.

The following search terms were used to identify relevant citations: (“depression” [Mesh] or “depressive disorder” [Mesh] or “depressive disorder, major” [Mesh] or “mood disorders” [Mesh]) and (“fractures, bone” [Mesh] or “fracture” or “bone fracture”) along with (“cohort studies” or “longitudinal studies” or “follow-up studies”). In addition, the reference lists of the original and relevant review articles were also assessed to further identify relevant studies. Papers published in English or Chinese were considered.

### Selection criteria

Studies were included in the meta-analysis based on the following inclusion criteria: (1) the cohort comprised non-institutionalized adults; (2) the exposure of interest was depression; (3) the outcome was fracture; (4) the risk estimates with the corresponding 95% confidence intervals (CI) of depression related to fracture were reported. Studies were excluded if (1) the study had a case control design or retrospective design; (2) Reviews, letters, commentaries were excluded; (3) Lack of any information that allowed to calculate effect estimates and corresponding 95% CI. For cohorts with several reports, we tried using data from non-overlapping follow-up periods of each report, or publications with the longest follow-up periods were selected.

### Data extraction and quality assessment

We extracted the following information from each eligible study: last name of the first author, year of publication, country where the study was performed, number of participants, characteristics of the participants (sex, age range, and mean age), follow-up time, depression measures, fracture type, and covariates adjusted in the multivariable analysis.

Quality assessment was performed according to the Newcastle–Ottawa scale (NOS) [13], which is a nine-point scale allocating points based on the quality of selection (0–4 points), comparability (0–2 points), and the outcomes of the study participants (0–3 points). In the NOS, poor, fair, and good quality were scored 0–3, 4–6, and 7–9, respectively. Two investigators (L.Q and Q.Y) independently performed the literature search, selected eligible studies and assessed their quality, and extracted data; disagreements or uncertainties were resolved via discussion with an additional investigator (X.X.Y).

### Statistical analysis

Hazard ratio (HRs) and their 95% CI were used to quantify the association between depression and fracture, and the reported relative risk (RRs) were considered equivalent to HRs. Any study results stratified by sex and fracture type were considered as independent reports. The heterogeneity of HRs across the studies was evaluated using the Cochran Q test (*P* value < 0.10 was considered an indication of statistically significant heterogeneity) and the *I*^*2*^ statistic (values of 25%, 50%, and 75% representing low, moderate, and high heterogeneity, respectively) [14, 15]. A fixed-effect model was used if no or low heterogeneity was detected; otherwise, the random-effects model was adopted [16].

Subgroup and sensitivity analyses were conducted to explore potential heterogeneity across studies, and the differences among subgroups were tested via meta-regression analysis. Publication bias was assessed via visual inspection of the funnel plot and using the Begg [17] and Egger tests [18]. The Duval and Tweedie nonparametric trim-and-fill method was used to adjust the potential publication bias [19]. Data were statistically analyzed with STATA version 11.0 (StataCorp, College Station, Texas, the USA). All statistical tests were two-sided with a 0.05 significance level.

### Patient involvement

No Patients were involved in determining the research question or the outcome measures or in developing plans for the design or implementation of the study. In addition, no patients were required to provide advice on the interpretation or writing of the results. There were no plans of disseminating the research results to the study participants or the relevant patient population.

## Results

### Literature search

**Fig. 1:**
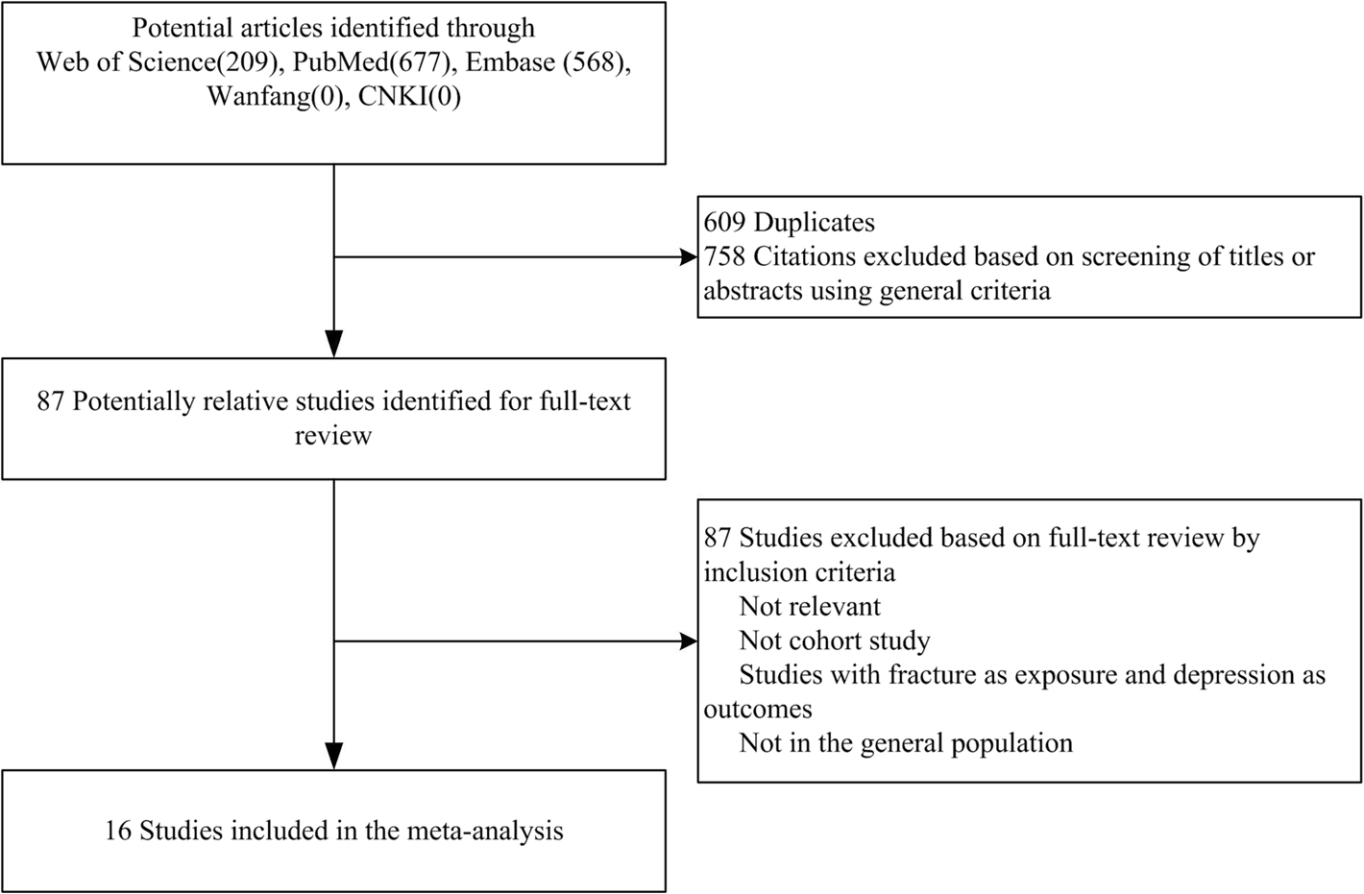
Flow chart of the Meta-analysis

Initially, we retrieved 209, 677 and 568 citations from Web of Science, PubMed, and Embase, respectively. After removing duplicates and reviewing the titles and abstracts, we identified 87 potentially relevant articles. After assessing the full text of articles that may be relevant, 16 eligible studies met the inclusion criteria and were finally included in our meta-analysis. The results of literature search and selection are presented in Fig. 1.

### Study characteristics

Additional file 1: Table S1 shows the main characteristics of the 16 cohort studies that were published between 1999 and 2017 and included in the present study. The quality assessment scores of all the studies ranged from 6 to 9, with an average score of 7.6. The size of the cohorts ranged from 482 to 139,110, with a total of 414,686 participants, of which 105,298 were men and 309,388 were women, and the follow-up durations ranged from 3 to 14 years. Of the 16 studies, most were from America (eight studies) or European countries (five studies). Meanwhile, one study was conducted in Australia [20] and two in Taiwan [8, 21]. Nine studies comprised both men and women, with four reporting results that were based on sex group, two studies included men only [22, 23], and five studies involved only women [20, 24–27]. In most of the studies, depression was assessed using self-reported symptom scales, and in three studies, the condition was confirmed based on the physician’s diagnosis [8, 21, 28]. With regard to fracture types, only five studies reported about any fracture [7, 20, 23, 26, 29], and only four studies were about hip fracture [8, 9, 27, 30]. Moreover, only two studies reported about nonvertebral fracture [22, 31], and one on vertebral fracture [21]. The remaining four studies were about two or more types of fracture [24, 25, 28, 32]. Adjusted HRs could be determined in all studies. The following confounding factors were adjusted: smoking status (nine studies), BMD (six studies), physical activity (five studies), and use of antidepressants (five studies).

### Association between depression and risk for fracture

**Fig. 2:**
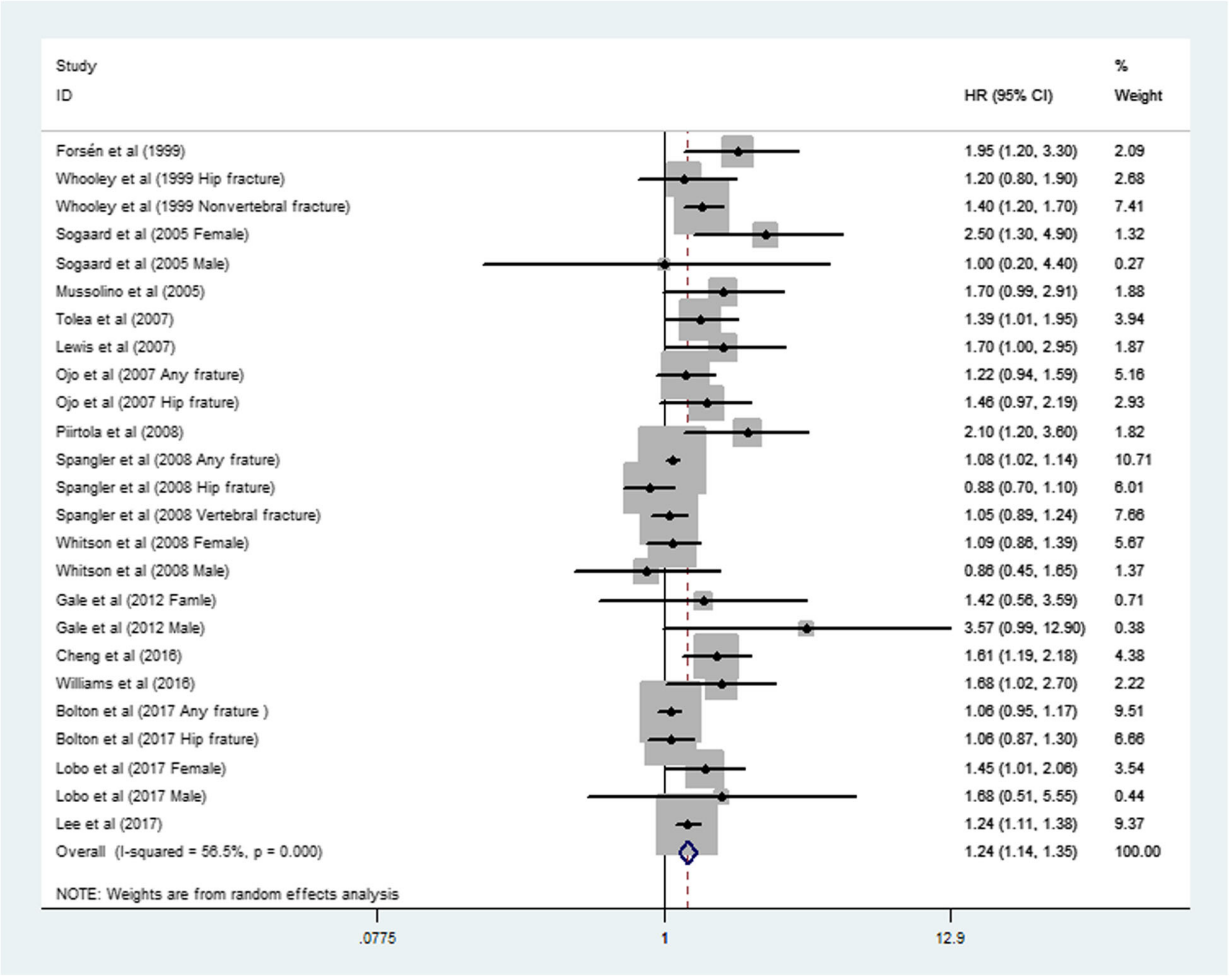
Forest plot of depression and the risk of fracture. Note: The summary estimates were obtained using a random-effects model. The diamond data markers indicated the pooled HRs. CI indicates confidence interval

Results from the random-effects meta-analysis of depression and the risk for fracture are presented in Fig. 2. Among the 25 reports from the 16 studies (which were stratified by sex and fracture type and were considered independent reports), most showed a positive association between depression and fracture incidence (i.e., HR > 1.00), of which 11 were statistically significant. The pooled HR was 1.24 (95% CI: 1.14–1.35), with substantial heterogeneity across studies (*P* = 0.000, *I*^*2*^ = 56.5%).

### Subgroup analyses

Table 1 shows the results from the subgroup analyses examining the stability of the primary results and to explore the latent source of potential heterogeneity. Low-to-moderate heterogeneities were observed in most subgroups. Depression was associated with an increased risk for fracture in all subgroups (HR > 1.00). The increased risk was more evident in the groups with a relatively small study sample (*n* < 5000). When applying study regional categories, the pooled HR for the studies conducted in European countries was higher (HR: 1.76; 95% CI: 1.44–2.17; *n* = 9) than that in America and Asia, with a statistically significant difference (*P* = 0.036). No significant difference was found between the groups in terms of other variables.

**Table 1:** Subgroup analyses on the association of depression and fracture risk

	No. of report^a^	HR (95% CI)	Q-Statistic	*P* value for heterogeneity	*I*^*2*^ (%)	*P* value between groups
Fracture Type						
Any fracture	9	1.16 (1.05–1.29)	15.74	0.073	42.8	0.834
Hip fracture	10	1.31 (1.08–1.59)	19.75	0.011	59.5	
Nonvertebral fracture	4	1.51 (1.23–1.85)	3.28	0.351	8.5	
vertebral fracture	2	1.16 (1.00–1.36)	2.7	0.100	63.5	
Sex						
Male	6	1.59 (1.11–2.29)	6.36	0.273	21.4	0.429
Female	12	1.23 (1.09–1.39)	29.87	0.002	63.2	
Mixed	7	1.22 (1.08–1.37)	12.83	0.046	53.2	
Mean age at baseline						
< 65	11	1.15 (1.05–1.26)	27.07	0.003	63.1	0.050
> =65	14	1.36 (1.22–1.50)	13.81	0.387	5.9	
Study region						0.036
America	14	1.13 (1.05–1.22)	22.95	0.042	43.4	
Europe, Australia	9	1.76 (1.44–2.17)	4.67	0.792	0	
Asia	2	1.36 (1.06–1.73)	2.53	0.112	60.5	
Duration of follow-up						
< 10	17	1.23 (1.10–1.38)	35.61	0.003	55.1	0.679
> =10	8	1.28 (1.11–1.47)	17.89	0.012	60.9	
Type of depression measure						
Self-reported scales	21	1.29 (1.16–1.44)	45.44	0.001	56.0	0.469
physician diagnoses	4	1.18 (1.03–1.36)	9.59	0.023	68.6	
Sample size						
< 5000	9	1.44 (1.25–1.66)	5.76	0.674	0.0	0.049
> =5000	16	1.18 (1.08–1.29)	38.78	0.001	61.3	
Control BMD in models						
Yes	10	1.15 (1.03–1.29)	19.87	0.019	54.7	0.109
No	15	1.36 (1.20–1.54)	29.9	0.008	53.2	
Control for antidepressants use						
Yes	7	1.19 (1.03–1.39)	12.52	0.051	52.1	0.513
No	18	1.28 (1.15–1.42)	42.31	0.001	59.8	
Control for smoking						
Yes	16	1.22 (1.08–1.38)	30.01	0.012	50	0.637
No	9	1.28 (1.14–1.44)	20.23	0.009	60.5	
Control for physical activity						
Yes		1.19 (1.01–1.41)	18.03	0.012	61.2	0.404
No		1.24 (1.44–1.35)	28.79	0.025	44.4	

Note: ^a^Four articles reported their results by sex group and four articles by type of fracture; there are 25 reports from 16 articles;*BMD* bone mineral density; *CI* confidence interval; *HR* Hazard ratio; Q-Statistic, Cochrane Q statistic; *I*^*2*^, the percentage of total variation due to heterogeneity among studies

### Publication bias

**Fig. 3:**
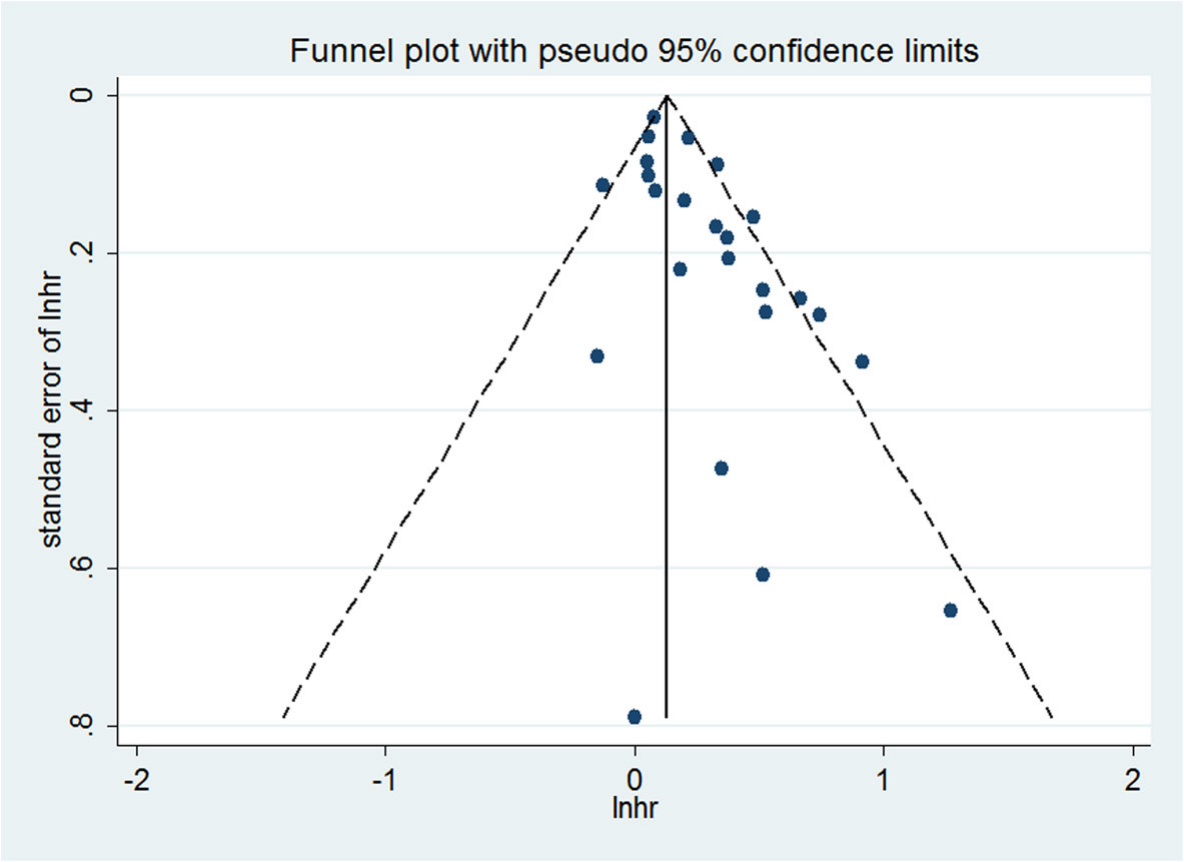
Funnel plot for studies on depression and fracture risk. Note: The horizontal line represents the summary effect estimates, and the dotted lines are pseudo 95% confidence intervals

**Fig. 4:**
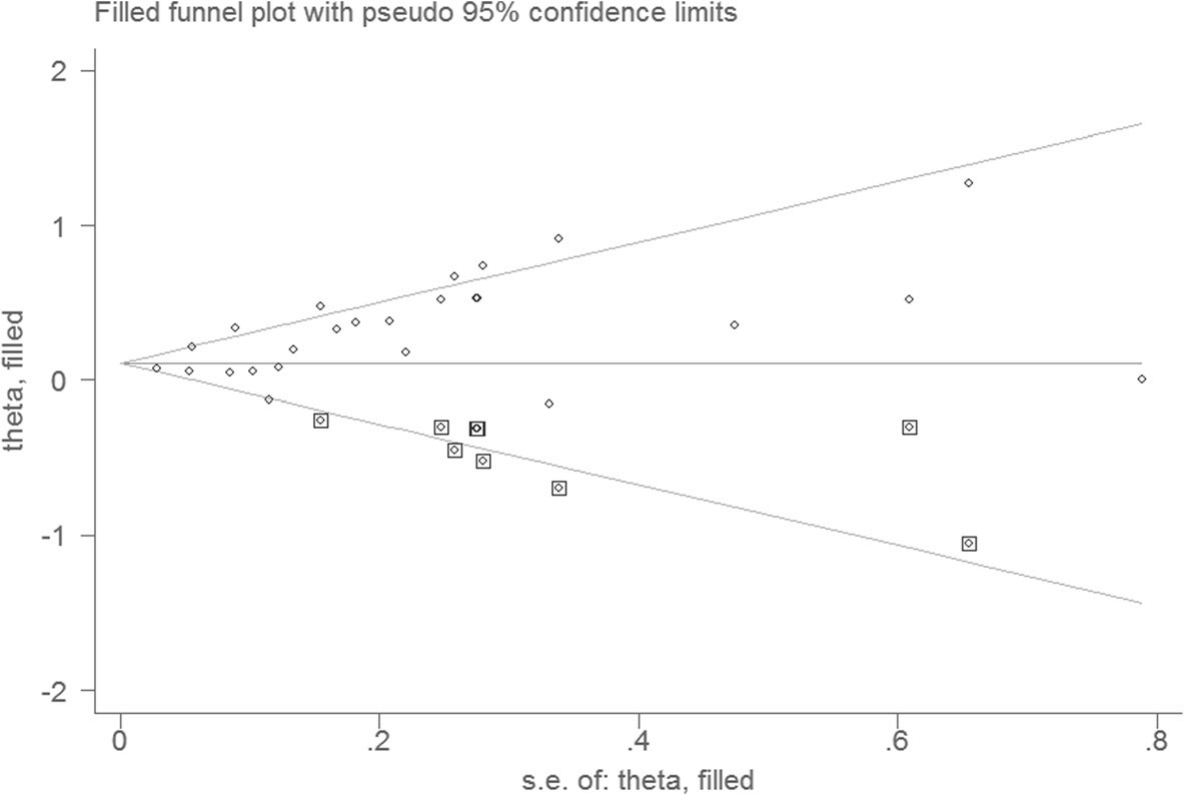
Filled funnel plot of HR from studies that investigated the association between depression and fracture risk. Note: The circles alone are real studies and the circles enclosed in boxes are “filled” studies. The horizontal line represents the summary effect estimates, and the diagonal lines represent pseudo- 95% CI limits

To examine the impact of a single study on the results, we omitted a study at each turn and pooled the results of the remaining studies. The pooled HR did not substantially change, ranging from 1.24 (95% CI: 1.14–1.35) to 1.29 (95% CI: 1.19–1.42). Visual inspection of the funnel plot revealed some asymmetry (Fig. 3). The Egger test suggested publication bias. However, the Begg test did not (Egger, *P* = 0.000; Begg, *P* = 0.018). We used the trim-and-fill method to assess the impact of any potential publication bias, and results showed that eight studies may be needed to obtain funnel plot symmetry for fracture (Fig. 4). By using the trim-and-fill method, the corrected HR was 1.14 (95% CI: 1.05–1.24; random-effects model, *P* = 0.003). Therefore, the pooled HR did not substantially change after the correction for potential publication bias.

## Discussion

Data from 16 cohort studies with 25 independent reports about 414, 686 participants were used, and our meta-analysis showed that depression was associated with a significantly increased risk for fracture. In addition, the associations remained statistically significant in the groups adjusted for fracture type, sex, study region, and other studies and characteristics of the participants; therefore, our results are robust and suggest that depression is prospectively associated with a significantly increases risk of fracture.

Our results showed that the pooled HR was 1.24 (95% CI: 1.14–1.35), which was slightly higher than that of a previous meta-analysis of 10 studies published before 2009 (HR: 1.17; 95% CI: 1.00–1.36) [10]. The current meta-analysis included 25 independent reports with sample sizes that are 4 times larger, which significantly enhanced statistical power and provided more accurate and comprehensive estimates of the association between depression and the risk for fracture. More importantly, compared with the previous meta-analysis, heterogeneity was thoroughly assessed, and subgroup analyses were conducted.

Our subgroups analyses identified an important finding. That is, the associations between depression and fractures varied between populations when subgroups analyses conducted by continents. The association was stronger in individuals from European countries than those from America and Asia. This may be attributed to the variation in health care systems in different geographic regions, availability of health services and other factors that are currently unknown. Since the studies included in the current meta-analysis were conducted in high-income countries(areas), such as those in Europe, North America, and Taiwan (research from Asian countries included only two studies conducted in Taiwan), these results should be cautiously generalized to developing countries. To apply this finding to a wider population, more studies must be conducted in other populations from Asia, Africa, and South America.

Depression may contribute to fracture via several possible mechanisms. First, depressive disorders are associated with the deregulation of the hypothalamic–pituitary–adrenocortical axis [33], chronic low-grade inflammatory response [34, 35], and increased oxidative and nitrosative stress [36]. These neuroendocrine and inflammatory effects caused by depressive disorders had implications for osteoporosis, which ultimately increased the risk for fracture [37, 38]. Second, some studies have shown that neuropathological lesions in certain regions of the brain in patients with depressive disorders can influence these patients’ balance, judgment, gait, and coordination, thus increasing the likelihood of falls, which in turn increases the risk for fracture [21, 39, 40]. Third, depression was associated with other major comorbidities, such as hypertension [41, 42] and diabetes [43]; these comorbidities were considered as risk factors for fracture, which were confirmed in two recent meta-analyses [44, 45]. Lastly, epidemiological findings showed that antidepressants, in particular selective serotonin reuptake inhibitors, may have direct effects on bone metabolism and decreased bone strength [46, 47], thereby increasing the risk for fracture [48]. However, the role of antidepressants should be cautiously interpreted because drug use can be a sign of severe depression, and numerous studies lacked information about the dose and duration of drug use.

This meta-analysis has several limitations. First, a moderate level of heterogeneity across studies was observed, which might result from the differences in the characteristics of the participants, sample sizes, depression measures, and statistical adjustments for potential confounders. Although moderate heterogeneities were still observed in some subgroups, the pooled HRs consistently showed positive associations in all subgroups and the prediction interval remained significant. Second, the funnel plot indicated a possible publication bias; however, the trim-and-fill method was used to correct the bias, which did not materially change the positive association. Third, we conducted only limited subgroups analyses because most original studies included in this meta-analysis did not adjust for other confounders such as social-economic status, medical comorbidities or ethnicity; information about these factors was not provided in most original reports. Finally, because the measurement of depression was mainly based on self-reported symptom scales, misclassification of exposure was inevitable, and this might bias the actual association between depression and fracture.

## Conclusion

In conclusion, this meta-analysis provides strong evidence that depression is significantly associated with an increased risk of fracture, particularly in individuals in Europe. Given the high prevalence of depression and osteoporotic fractures in the general population, the observed association between depression and fractures have substantial implications, both clinical and preventive. Mental health is closely related to bone health. It is greatly important for primary care practitioners and mental health care workers to take depression account into the prevention and clinical treatment of osteoporotic fractures.

## Additional file

Additional file 1: **Table S1.** Characteristics of studies included in the meta-analysis. (DOCX 18 kb)

## Abbreviations

BMD: Bone mineral density.
CI: Confidence interval
HRs: Hazard ratios
NOS: the Newcastle–Ottawa scale
RRs: Relative risks
