# Supplementary material for: Association between depression and the risk for fracture: a meta-analysis and systematic review
Source: BMC Psychiatry. 2018 Oct 17;18:336. doi: 10.1186/s12888-018-1909-2 (PMC6192066; doi:10.1186/s12888-018-1909-2)
Supplement: Supplementary file 1 — Table S1. Characteristics of studies included in the meta-analysis. (DOCX 18 kb) [file 12888_2018_1909_MOESM1_ESM.docx]

| Authors/years  **Table S1 Characteristics of studies included in the meta-analysis** | Participants/ Male(%) | Baseline age | Follow-up years | Depression Measures | Outcomes | Variables controlled | Quality score |
| --- | --- | --- | --- | --- | --- | --- | --- |
| Williams et al, 2016; Australia | 858 /0 | range 35–60; mean 47.5 | 10 | SCID-I/NP | Any fracture | Age, education, BMI, smoking, physical activity, BMD, prior fracture, falls and bone active medications, and use of antidepressants | 7 |
| Cheng et al,  2016; China | 139110/38 | range ≥46.5;  mean 46.5 | 14 | physician diagnosis | Hip fracture | Sex, age, urbanization, osteoporosis, and use of antidepressants | 8 |
| Gale et al,  2012; England | 2137/51 | range 59-73;  mean 60.5 | 5.6 | HADS | Any fracture | Age, physical activity, alcohol intake, smoking, calcium intake and prior fracture | 8 |
| Tolea et al,  2007; America | 1350/0 | range ≥65;  mean 75 | 7 | CES-D | Any fracture | Demographic variables, alcohol intake, diabetic, osteoporosis at baseline, number of pregnancies, has hysterectomy, BMD, and poor/fair self-rated health | 8 |
| Lewis et al,  2007; America | 5995/100 | range ≥65;  mean ≥65 | 4.1 | SF-12 | Nonvertebral fracture | Clinical site, race/ethnicity, BMD, fracture at or after age 50, Age 80 years, any fall in past year, use of antidepressants , unable to complete any narrow walk trial | 9 |
| Whooley et al, 1999; America | 7414/0 | range≥65;  mean 73.37 | 6 | GDS | Hip fracture;  Nonvertebral fracture | Age, marital status, education, BMD | 8 |
| Ojo et al,  2007; Mexico | 2621/41 | range≥65;  mean 72.45 | 7 | CES-D | Hip fracture;  Any fracture | Demographic variables, smoking, medical conditions, cognitive status, high depressive symptoms, vision function, BMI and lower body function score | 8 |
| Forsén et al,  1999; Norway | 18 612/0 | range 50-101;  mean 66 | 3 | MDI | Hip fracture | Age, mental distress, smoking, physical activity, and impairment because of physical illness | 8 |
| Sogaard et al, 2005; Norway | 12270/50 | range 20-54; mean<60 | 7 | MDQ | Nonvertebral fracture | Age, marital status, smoking and alcohol intake | 6 |
| Lobo et al,  2017; Spain | 4660/42 | range >55;  mean 73.4 | 16 | GMS | Hip fracture | Civil status, illiterate, smoking, alcohol intake ,and BMI | 7 |
| Whitson et al, 2008; Cannada | 5827/72 | range >50;  mean 66 | 5 | MHI-5 and MCS | Any fracture | Age, BMD, steroid use, smoking, history of falls, prior fracture, and use of antidepressants | 7 |
| Bolton et al,  2017; Canada | 68 730/9 | range >40;  mean 64.2 | 18 | physician diagnosis | Any fracture;  Hip fracture | Sex, age and use of antidepressants | 8 |
| Spangler et al, 2008; USA | 82410 /0 | range 50-79;  mean | 7.4 | CES-D | Any fracture;  Hip fracture;  Vertebral fracture | Age, weight, height, ethnicity, years since menopause, physical activity, smoking, analgesic or narcotics, BMD, and previous fracture | 8 |
| Piirtola et al,  2008; Finland | 482/100 | range 65-97;  mean 73 | 12 | ZSDS | Any fracture | Age, handgrip strength, occurrence of a previous fracture after 45 years of age and compression of thoracic or upper lumbar vertebrae | 6 |
| Mussolino et al, 2005; USA | 6195 /46 | range 25-74;  mean 49 | 22 | GWB-D | Hip fractures | Age, sex, race, smoking, alcohol intake, and physical activity | 7 |
| Lee et al  2017; China | 56015/40 | range 29-51;  mean 39 | 11 | physician diagnosis | Vertebral fracture | Age, sex, degree of urbanization, monthly income | 9 |

Note: Charlson comorbidity index(CCI), SCID-I/NP, semi-structured clinical interview; HADS, Hospital Anxiety and Depression Scale; CES-D, Center for Epidemiologic Studies Depression Scale; SF-12, Self-reported mood question from12-item short-form health survey; GDS, Geriatric Depression Scale; MDI ,mental distress index; GMS, Geriatric Mental State scale; MHI-5, the mental health inventory-5 scale; MCS, the mental component score; ZSDS, Zung Self-rating Depression Scale; GWB-D, The General Well-Being Schedule; BMD, bone mineral density.
